# New Appointments at the Faculty of Medicine of Paris

**Published:** 1859-03

**Authors:** 


NEW APPOINTMENTS AT THE FACULTY OF MEDICINE OF PARIS.—The Chair of Anatomy, rendered vacant by the retirement of M. Denonvilliers, has been filled by the appointment of M. Jarjavay. M. Gosselin succeeds M. Cloquet as Professor of Surgical Pathology.

